# Draft genome sequence of *Bacillus altitudinis* strain MSH-24 isolated from rhizosphere soil of *Nypa fruticans* (Golpata plant) in the mangrove forest in Bangladesh

**DOI:** 10.1128/mra.00701-26

**Published:** 2026-09-30

**Authors:** Didar Hossain, Md. Saddam Hossain

**Affiliations:** 1 Department of Industrial Biotechnology, Faculty of Biotechnology and Genetic Engineering, Chattogram Veterinary and Animal Sciences University 130057, Chattogram, Bangladesh; 2 Department of Genomics and Bioinformatics, Faculty of Biotechnology and Genetic Engineering, Chattogram Veterinary and Animal Sciences University 130057, Chattogram, Bangladesh; University of Manitoba, Winnipeg, Canada

**Keywords:** draft genome, *Bacillus altitudinis*, genome assembly, genome annotation, biopesticides

## Abstract

We report the draft genome of *Bacillus altitudinis* MSH-24 isolated from rhizosphere soil of *Nypa fruticans* in Bangladesh. Illumina NovaSeq sequencing generated a 3.9 Mb assembly (41.08% GC) in 22 contigs, revealing genes associated with possible iron acquisition, stress tolerance, sporulation, and germination, highlighting the strain’s environmental adaptability and metabolic versatility.

## ANNOUNCEMENT

The genus *Bacillus* comprises gram-positive, spore-forming bacteria commonly associated with environmental habitats, including soil, water, and plant-associated niches (1). Strain MSH-24 was isolated from a rhizosphere soil sample of *Nypa fruticans* (Golpata plant) collected from a mangrove forest in Khulna, Bangladesh (22°27′30.2″ N, 89°23′49.9″ E). The isolation of strain was obtained to explore its potential as a naturally occurring, environmentally safe biopesticide, highlighting the importance of characterizing indigenous microbial resources (2). The soil sample was transported on ice and processed immediately. Briefly, 1 g of soil was suspended in 9 mL of sterile distilled water, vortexed to release bacterial cells, and heated at 80°C for 10 min. Tenfold serial dilutions (10^−1^ to 10^−5^) were prepared, and 100 μL of each dilution was plated on nutrient agar (HiMedia, India) in sterile 100 mm petri dishes. Plates were incubated at 35°C for 24 h. Individual colonies were purified by repeated streaking, grown for 24 h, and stored at 4°C until further analysis.

Genomic DNA was extracted using the TIANamp bacterial DNA kit (TIANGEN Biotech, Beijing, China), and quality and quantity were evaluated using a UV/Vis BioPhotometer (Eppendorf, Germany). Paired-end sequencing libraries were prepared using the Nextera DNA Flex Library Preparation Kit (Illumina, USA) and sequenced on an Illumina NovaSeq 6000 platform to generate 150-bp paired-end reads (2 × 150 bp) at Novogene, China. Read quality was assessed with FastQC Galaxy v0.74 (3), and adapters and primers were removed using Trim Galore Galaxy v0.6.7 (4). Trimmed reads were assembled *de novo* with SPAdes Galaxy v3.15.5 (5), and assembly quality was evaluated using QUAST Galaxy v5.2.0 (6). The draft genome was primarily annotated via NCBI PGAP v6.10 (7), respectively. Default parameters were used unless specified. Genome assignment was performed using GTDB-Tk v2.6.1 (ANI 98.4%, assembly coverage 90.08%) (8), and genome completeness and contamination were assessed using CheckM v1.2.4 (9).

The draft genome of strain MSH-24 is 3,875,739 bp, assembled into 22 contigs, with an N50 of 679,144 bp, L50 of 3, and GC content of 41.08%. Genes involved in ABC-type Fe³^+^-siderophore transport indicate potential for iron acquisition (10). Flagellar genes (*fliE*/*F*/*G*/*I*/*J*/*L*/*M*/*Y*/*Z*/*P*/*Q*/*R*) suggest motility and plant-root colonization potential (11). Genes encoding acetolactate synthase, α-acetolactate decarboxylase, and 2,3-butanediol dehydrogenase indicate potential production of acetoin and 2,3-butanediol, which may support plant-microbe interactions (12). The presence of *pgsA* and *pgsB* further suggests biofilm formation and root colonization (13). Sporulation and germination genes (*spo0A*/*F*/*B*, *kin*, *sigF*/*E*/*G*/*K*, *gerD*, *gerP*, *cwlJ*, *sleB*, and *cwlD*) support environmental persistence and survival (14–16). Collectively, these features highlight the environmental resilience and plant-associated potential of *Bacillus altitudinis* MSH-24, supporting its candidacy as a potential biopesticide. Experimental and field-scale validation is required to confirm these functions (Table 1).

**TABLE 1:** Genomic features of *Bacillus altitudinis* strain MSH-24 isolated from rhizosphere soil of *Nypa fruticans* (Golpata plant) in the Sundarbans mangrove forest in Bangladesh^*a*^

Genome features	MSH-24
Number of reads	9,755,756
Assembled genome size (bp)	3,875,739
GC content (%)	41.08
Coverage (×)	34
Coarse consistency (%)	98.2
Fine consistency (%)	96.5
Genome completeness (%)	99.12 (CheckM)
Contamination (%)	0
L50	3
N50 value (bp)	679,144
N ratio	0.0
Number of contigs	22
Contig density	89.0
CDSs (total)	3,972
Genes (total)	4,048
Genes (coding)	3,947
CDSs (with protein)	3,947
CDSs (without protein)	25
Genes	76
rRNAs	1, 1, 1 (5S, 16S, 23S)
Complete rRNAs	1, 1, 1 (5S, 16S, 23S)
tRNAs	68
tmRNAs	1
ncRNAs	5
ncRNA regions	62
Pseudogenes (total)	25

^*a*^ CDSs, coding sequences.

## Data Availability

The whole genome shotgun project of *Bacillus altitudinis* strain MSH-24 has been deposited in GenBank under BioProject (accession number PRJNA224116) and the NCBI Sequence Read Archive (accession number SRX34906946). The corresponding BioSample accession number is SAMN46137717. The version described in this paper is version NZ_JBLGDX000000000.1.
